# Feeling better informed and more confident to speak up in the operating room: team effects of a structured intraoperative briefing intervention – a before-after study

**DOI:** 10.1136/bmjoq-2025-003989

**Published:** 2026-10-20

**Authors:** Franziska Tschan, Eliane Timm-Holzer, Sandra Keller, Jasmin Zimmermann, Norbert K Semmer, Simon A Huber, Martin Hübner, Simon Wrann, Stefan Gutknecht, Gian Andrea Prevost, Daniel Candinas, Nicolas Demartines, Markus Weber, Guido Beldi

**Affiliations:** 1 Institute of Work and Organizational Psychology, University of Neuchâtel, Neuchâtel, Switzerland; 2 Department of Visceral Surgery and Medicine, Inselspital University Hospital Bern, Bern, Switzerland; 3 Institute of Work and Organizational Psychology, University of Bern, Bern, Switzerland; 4 Department of Visceral Surgery, CHUV, Lausanne, Switzerland; 5 Department of Surgery, Municipal Hospital Triemli, Zürich, Switzerland; 6 Chirurgie, Kantonsspital Graubünden, Chur, Switzerland; 7 Department of Surgery, HFR Fribourg Cantonal Hospital, Fribourg, Switzerland; 8 Faculty of Science and Medicine, University of Fribourg, Fribourg, Switzerland

**Keywords:** Teamwork, Checklists, Communication, Surgery, Teams

## Abstract

**Background:**

Effective intraoperative teamwork depends on shared situation awareness (SA) and the ability to express concerns without hesitation (ease of speaking up, ESU). However, operating room (OR) team members often lack critical updates during surgery and may hesitate to speak up. This study evaluated the impact of the StOP?-protocol—a structured intraoperative briefing previously linked to reduced postoperative complications—on informedness (a key aspect of SA) and ESU.

**Methods:**

In a multicentre before-after study in three Swiss hospitals, lead surgeons implemented the StOP?-protocol (St: current status; O: next objectives; P: potential problems; ?: invitation for team input). After each operation, team members rated how well informed they felt and their ESU. Primary outcomes were differences in perceived informedness and perceived ESU between baseline and intervention phases, analysed across roles with covariance adjustment.

**Results:**

A total of 371 operations were analysed (165 baseline, 206 intervention). The intervention significantly improved perceived informedness for anaesthesia providers (B=0.73, 95% CI 0.47 to 0.98) and circulators (B=0.56, 95% CI 0.32 to 0.81), and improved ESU for scrub technicians (B=0.37, 95% CI 0.09 to 0.66), anaesthesia providers (B=0.34, 95% CI 0.08 to 0.61) and circulators (B=0.28, 95% CI 0.03 to 0.54). Effects were strongest among professionals distant from the operative field. No significant changes were observed for residents.

**Conclusion:**

A brief, structured, surgeon-led intraoperative briefing improved perceived informedness and ESU in OR teams. By proactively engaging all team members, the StOP?-protocol addresses key barriers to safe intraoperative teamwork and complements existing checklist-based approaches. Its implementation may strengthen teamwork-related processes in surgery.

**WHAT IS ALREADY KNOWN ON THIS TOPIC**

- Operating room team members often feel underinformed during operations, and many hesitate to raise concerns. Existing interventions have mainly focused on preoperative and postoperative checklists, with little attention to intraoperative briefings.

**WHAT THIS STUDY ADDS**

- Implementing the StOP?-protocol, a brief, surgeon-led structured update during the operation, improved both perceived informedness and ease of speaking up across several professional roles within the operating room team.

**HOW THIS STUDY MIGHT AFFECT RESEARCH, PRACTICE OR POLICY**

- This study provides evidence that structured intraoperative briefings can complement established safety checklists and may be a practical tool to strengthen team informedness and ease of speaking up and thus promote teamwork processes relevant to surgical safety.

## Introduction

 Optimal collaboration among operating team members is crucial for surgical performance, alongside technical skills.^1–3^ Intraoperative cooperation and communication issues can lead to prolonged operations,^4^ increased errors and compromised technical performance,^5–8^ potentially threatening patient safety.^9–12^

One condition for good team performance, as evidenced in various fields^13 14^ including surgery,^15–17^ is situation awareness (SA).^18 19^ SA refers to the (shared) understanding of tasks and their context, particularly emphasising dynamic aspects. Endsley^19^ succinctly describes SA as ‘knowing what is going on around you’ (p. 2). SA comprises three levels: perception, understanding and projection. *Perception* relates to perceiving ongoing events (eg, observing the surgeon’s actions); *understanding* entails comprehending their meaning (eg, recognising that the surgeon is resecting X) and *projection* involves anticipating future developments and the corresponding action requirements (eg, predicting the surgeon’s next move). Thus, SA allows team members to anticipate the unfolding situation,^20 21^ thus facilitating coordination. To foster and maintain SA during an operation, team members need up-to-date information about the current status, ongoing developments, potential problems and any strategy changes.^17 22^

The surgeon responsible for an operation has a central role in both planning and executing the operation,^23 24^ and thus holds critical information not always known to the other members of the surgical team.^17^ A key non-technical skill for surgeons is to provide critical information to the team,^25 26^ as more information exchange during operations is related to improved patient outcomes.^27 28^ However, a common concern among operating room (OR) team members is that they do not receive sufficient information from surgeons during operations.^29^ This is especially challenging for remote team members like anaesthesia providers and circulators, who depend on updates from the surgeon for SA, lacking direct visual oversight of the operative field. However, surgeons direct <1% of their communication to the entire team, while predominantly engaging in dyadic communications with other surgeons and scrub technicians,^30^ who are directly involved at the operating table.

Another integral aspect of good team performance is speaking up, described as voicing ideas or concerns, including asking critical questions and pointing out errors.^31^ In medical teams, ease of speaking up (ESU) was linked with error prevention, error identification and correction.^31^ However, OR team members may hesitate to speak up because it may entail interpersonal risks, such as facing criticism or ridicule.^32–34^ To enhance ESU, explicitly encouraging team members to ask questions or voice concerns is recommended,^35–37^ particularly from team leaders.^38 39^

Interventions promoting information exchange and teamwork in the OR are promising with regard to patient outcomes.^40–43^ One such intervention is the StOP?-protocol, a short intraoperative briefing initiated and led by the responsible surgeon at least once per operation.^44^ Its goal is to update the entire team with information regarding the operation’s process, adaptations in strategy, and potential upcoming challenges and problems. In addition, it includes an explicit encouragement for the team members to participate and to speak up. Previous research has shown that the StOP? improves patient outcomes, including a reduction in mortality.^44^ However, its effect on surgical team members’ sense of being informed (as part of SA) and their willingness to speak up (ESU) remain unknown. The current study explores effects of the StOP? intervention on being informed and ESU of OR team members.

The StOP?-protocol facilitates the exchange of case-related information during the operation and is assumed to enhance SA of OR team members. The main objective of this study was to compare team members’ level of informedness about what the surgeon was doing during the operation and the plans and strategies behind these actions, before and after the introduction of the StOP?-protocol. The secondary objective was to assess changes regarding reported ESU by surgical team members following the introduction of the StOP?-protocol.

## Materials and methods

This observational study was carried out alongside a larger multicentre, before-after study investigating the effect of the StOP?-protocol on patient outcomes across four hospitals.^44^ Two major academic hospitals in Switzerland (general surgery) and one regional hospital (general and vascular surgery) participated. In the fourth hospital included in the broader project, baseline data could not be collected. All hospitals also used the WHO surgical safety checklist pre-incision.

Operations were eligible if they were elective procedures expected to last longer than 60 min and if a research team member could be present in the OR to distribute and collect the questionnaires. Cases were excluded when the patient had an active surgical-site infection or had undergone another procedure at the same site within the previous 30 days. During the baseline period, we included a representative mix of elective operations typical for each hospital. For the intervention phase, we sought to approximate the same composition of procedures. Online supplemental table S1 shows surgery characteristics and baseline vs intervention comparisons. A total of 371 operations were included, 165 during baseline and 206 during the intervention period.

### Intervention

The 9-month intervention period was preceded by a 9-month baseline and a 4-week introductory period, with a staggered start between hospitals. The intervention involved implementing the StOP?-protocol during operations (see box 1). The briefing was initiated by the responsible surgeon who paused the operation and asked all OR team members to approach the table.

Box 1 **The StOP?-protocol: structure of the intraoperative briefing**

**StOP? stands for**

St: status: the surgeon explains the current situation.

O: objectives: the surgeon states the goals and next steps.

P: problems: the surgeon mentions potential problems that may arise.

?: questions: the surgeon invites questions, comments or concerns from team members.

In order to mitigate attentional overload of the surgeon and prevent untimely interruptions, the timing of the briefing was chosen by the surgeon and adapted to the unique requirements of each surgical procedure. It took place at natural transition points within the operation (eg, prior to a critical phase or before implementing a strategy change).^45^ Briefings typically lasted between 30 and 90 s. For more detailed information, see the publication concerning patient outcomes^44^ and online supplemental figure S1.

### Procedure

Before the baseline period, the surgical teams received general study information. Before the intervention period, specific training concerning the StOP?-protocol, involving presentations, videos and written materials, was conducted. During one-to-one instructional interviews, surgeons identified natural breakpoints for briefings for their specific operations and provided their consent for participation in the study. During the intervention period, posters were placed on doors, near hand-washing facilities and in each OR as reminders. Monthly adherence feedback was given during the intervention.

A research team member was present in the OR for operations in the study subset. Before leaving the OR, each OR team member received a questionnaire from the research team member present. Anaesthesia professionals completed their questionnaires shortly before the end of operations to prevent interference with the emergence phase.

All operating team members received oral information during dedicated meetings and written information about the study prior to data collection. When questionnaires were distributed, it was explicitly stated again that participation was voluntary and that individuals could decline without any consequences. Completion of the questionnaire was considered to constitute implied consent. Confidentiality was ensured, as researchers collected and boxed questionnaires without viewing responses, and no demographic data were collected.

Response rates for questionnaires were 98.9% for responsible surgeons, 100% for second surgeons, 95.5% for residents, 99.5% for scrub technicians, 99.2% for anaesthesia providers and 99.2% for circulators. Note that second surgeons and residents were not present in all operations; response rates therefore reflect questionnaire completion when a second surgeon or resident was present. Questionnaires were brief to reduce respondent burden.

### Outcome measures

Outcome measures were based on self-report. *Feeling informed* was assessed using two questions: ‘To what extent were you always informed about the plans and strategies the surgeons had in mind?’ and ‘To what extent were you always informed about what the surgeons were doing at the very moment?’ Responses ranged from (1) *absolutely not informed* to (7) *completely informed*. These questions were adapted from the SART SA technique of Taylor, 1990, cf,^46^ for details, see online supplemental materials. The questions were combined into an index (Cronbach’s alpha: 0.87 for residents, 0.88 for scrubs, 0.86 for anaesthesia, 0.92 for circulators). These questions were not included in the surgeon’s questionnaires.

ESU was assessed with the question ‘During this operation, could you have asked questions or raised issues at any time?’ adapted from the Speaking Up Climate for Professionalism (SUC-prof)^47^; for details see online supplemental materials. Responses ranged from (1) *without any hesitation* to (7) *with a great deal of hesitation*. This question was reverse scored for analysis, with higher numbers indicating greater ESU.

#### Other measures

*Difficulty of surgery* was assessed by the responsible surgeon asking, ‘How challenging was this surgery for you?’ from 1 to 7 (1=*very easy, routine;* 7=*very challenging*). *Operative approach* was categorised as minimally invasive (0) or open (1), with converted operations coded as open.

*Adherence during the intervention phase* was assessed with the item ‘Was a StOP?-protocol carried out?’ Adherence was assumed when at least two members of the surgical team independently reported that a StOP?-protocol had been carried out. Based on this criterion, adherence was 96.1%.

### Data analysis

The sample size was determined by eligibility criteria, and post-hoc power analysis was not conducted, following current recommendations.^48^ Internal consistency was assessed using Cronbach’s alpha.^49^ Questionnaire responses were averaged within professional roles if multiple individuals of the same role were present. Descriptive statistics, including means, SD and proportions were calculated. Between-group differences in operation type and operative access before and during the intervention were assessed using χ^2^ tests, differences in perceived difficulty were assessed with independent sample t-tests.

For each professional role, differences in perceived informedness and ESU at baseline and during the intervention were analysed using analyses of covariance (ANCOVAs), adjusting for hospital, operative access and case difficulty.

To account for potential clustering of observations within surgeons, we additionally conducted linear mixed-effects models with random intercepts for surgeons as a robustness check.

Associations between difficulty ratings and operative access with feeling informed and ESU, along with interaction effects, were assessed through ANCOVA models, adjusting for intervention and hospitals. Interaction effects between the intervention and access, as well as the intervention and difficulty, on feeling informed and ESU were based on separate ANCOVAs. Statistical significance level was set at p<0.05. Data were analysed using SPSS V.28,^50^ figures were generated with JASP.^51^

## Results

There were no significant differences observed in the case mix, proportion of minimally invasive procedures or difficulty of operations between the baseline (165 operations) and intervention period (206 operations), see online supplemental table S1. Feeling informed and ESU correlated moderately (between 0.35 and 0.43) within professions, supporting that they measure different constructs (see online supplemental table S2).

We first report results regarding the changes in feeling informed and ESU during the intervention as compared with baseline, followed by additional analyses investigating the relationship between surgical difficulty and operative access on feeling informed and ESU.

### Effects on feeling informed

Tables 1 and 2 and figure 1 present unadjusted and adjusted results on feeling informed for residents, scrubs, anaesthesia providers and circulators. While there was no significant improvement for residents and scrubs, anaesthesia providers and circulators reported feeling significantly better informed during the intervention compared with baseline.

**Table 1:** Unadjusted differences between baseline and intervention for feeling informed and ease of speaking up for operating room team members

	Baseline	Intervention	t (df)	P value
	M (SD)	M (SD)		
Feeling informed (1–7)				
Residents (n=297)	6.32 (0.89)	6.34 (1.01)	−0.217 (295)	0.828
Scrub technicians (n=369)	5.97 (1.04)	6.09 (1.05)	−1.152 (367)	0.250
Anaesthesia (n=368)	4.83 (1.29)	5.52 (1.17)	−5.316 (366)	<0.001
Circulators (n=368)	5.26 (1.27)	5.78 (1.19)	−4.050 (366)	<0.001
Ease of speaking up (1–7)				
Residents (n=295)	6.12 (1.39)	5.99 (1.68)	0.669 (293)	0.504
Scrub technicians (n=364)	5.93 (1.53)	6.26 (1.25)	−2.222 (310.9)	0.027
Anaesthesia (n=364)	5.76 (1.47)	6.06 (1.11)	−2.134 (294.9)	0.034
Circulators (n=366)	5.82 (1.33)	6.09 (1.22)	−2.060 (364)	0.040

M, mean; P, significance level.

**Figure 1:**
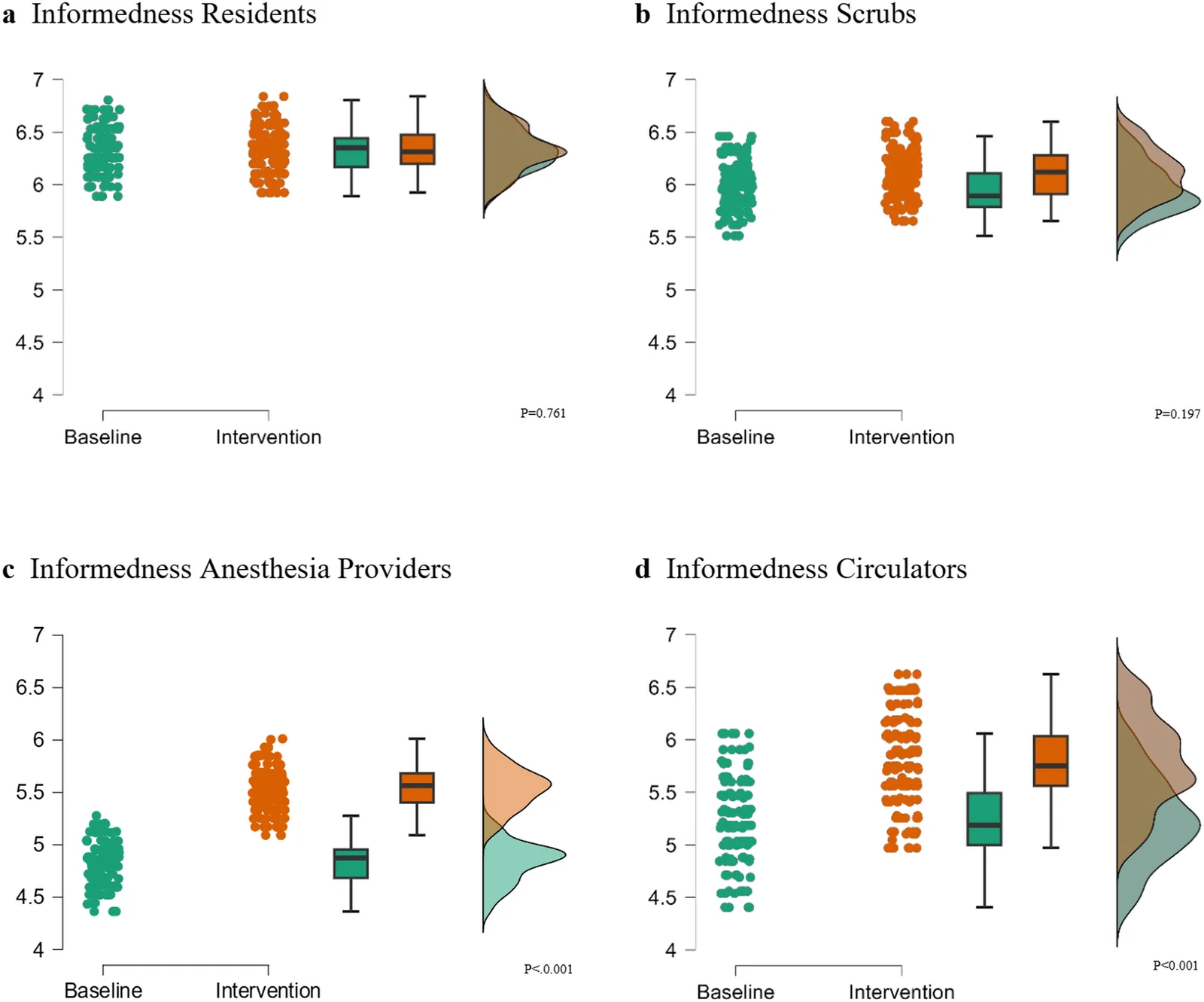
Perceived informedness at baseline and intervention for each professional group. Values are predicted from ANCOVA models adjusted for hospital, operative access and surgical difficulty. Scores range from 1 (not at all informed) to 7 (completely informed). Error bars represent 95% CIs. ANCOVA, analysis of covariance.

**Table 2:** Covariate-adjusted differences between baseline and intervention for feeling informed and ease of speaking up for OR team members. Results are adjusted using ANCOVA models, controlling for hospital, access and case difficulty

	Baseline	Intervention	Difference intervention vs baseline			
	M (95% CI)	M (95% CI)	F (df=1)	P value	B	95% CI diff
Feeling informed (1–7)						
Residents (n=291)	6.31 (6.15 to 6.47)	6.35 (6.20 to 6.50)	0.092	0.761	0.03	−0.19 to 0.26*
Scrub technicians (n=365)	5.96 (5.80 to 6.12)	6.1 (5.96 to 6.24)	1.673	0.197	0.14	−0.07 to 0.35*
Anaesthesia (n=364)	4.83 (4.64 to 5.01)	5.55 (5.38 to 5.72)	31.896	<0.001	0.73	0.47 to 0.98†
Circulators (n=364)	5.24 (5.06 to 5.42)	5.8 (5.64 to 5.96)	20.613	<0.001	0.56	0.32 to 0.81*‡
Ease of speaking up (1–7)						
Residents (n=291)	6.07 (5.82 to 6.33)	6.06 (5.82 to 6.30)	0.003	0.955	−0.01	−0.36 to 0.34‡
Scrub technicians (n=364)	5.91 (5.70 to 6.12)	6.28 (6.09 to 6.47)	6.618	0.010	0.37	0.09 to 0.66‡
Anaesthesia (n=364)	5.75 (5.55 to 5.95)	6.09 (5.92 to 6.27)	6.244	0.012	0.34	0.08 to 0.61*
Circulators (n=362)	5.81 (5.62 to 6.0)	6.09 (5.92 to 6.26)	4.666	0.031	0.28	0.03 to 0.54*‡

Superscripts indicate significant covariate effects.

* difficulty.

† operative access.

‡ hospital.

ANCOVA, analysis of covariance; B, adjusted mean difference; df, degrees of freedom; F, F-statistic; M, mean, adjusted for hospital, operative access (open vs minimally invasive) and surgeon-rated difficulty of operation; P, significance level.

Additional analyses explored the influence of difficulty and operative access on feeling informed. Operations perceived as more difficult were associated with a lower level of feeling informed for residents (t=−2.626, p=0.009), scrub technicians (t=−2.962, p=0.003) and circulators (t=−3.811, p<0.001), but not for anaesthesia providers (t=−1.932, p=0.054). Moreover, minimally invasive operations were associated with lower feeling informed scores for anaesthesia providers (t=−2.576, p=0.010), but not for residents (t=0.728, p=0.467), scrub technicians (t=1.309, p=0.191) or circulators (t=0.990, p=0.323); see online supplemental table S3 and online supplemental figure S2.

The impact of the intervention on the relationship between difficulty/access and feeling informed was not significant for residents, scrub technicians or anaesthesia providers. However, circulators reported a greater increase in feeling informed for open as compared with minimally invasive operations during the intervention, as indicated by a significant interaction effect, see online supplemental figure S3.

### Effects on ESU

ESU was generally rated as high, with mean scores between 5.8 and 6.3 on a seven-point scale. Residents consistently reported high ESU during both baseline and intervention. However, scrub technicians, anaesthesia providers and circulators reported significantly higher ESU during the intervention compared with the baseline period (tables 1 and 2, figure 2).

**Figure 2:**
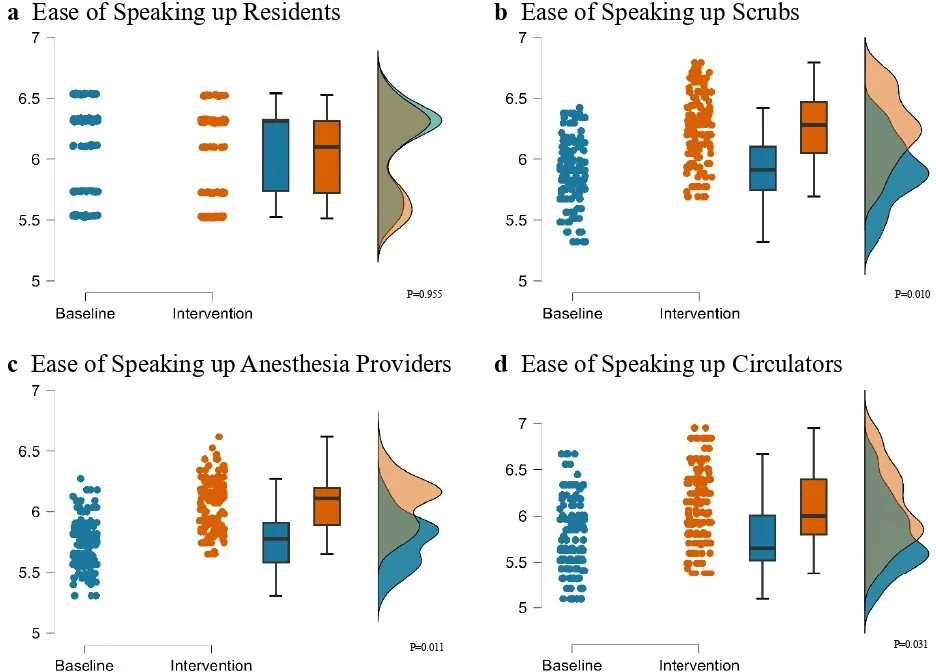
Perceived ease of speaking up at baseline and intervention for each professional group. Values are predicted from ANCOVA models adjusted for hospital, operative access and surgical difficulty. Scores range from 1 (with a great deal of hesitation) to 7 (without any hesitation). Error bars represent 95% CIs. ANCOVA, analysis of covariance.

Additional analyses (online supplemental table S4 and online supplemental figure S4) found no significant association between more difficult operations and ESU for residents (t=0.049, p=0.961) and scrub technicians (t=1.728, p=0.085). However, anaesthesia providers (t=−2.093, p=0.037) and circulators (t=−2.642, p=0.009) reported lower ESU during more difficult operations. Operative access was not significantly associated with ESU for any profession. We probed interaction effects between the intervention and access, as well as between the intervention and difficulty on ESU; none of the interactions was significant.

### Robustness analyses accounting for clustering

For all intervention effects reported above, we conducted additional linear mixed-effects models to account for clustering of observations within surgeons. Results were consistent with the ANCOVA findings across all outcomes for all professional roles. Accounting for clustering did not change the substantive conclusions (online supplemental table S5).

## Discussion

### Feeling informed and ESU during baseline and intervention

This study examined whether introducing the StOP?-protocol, a structured intraoperative briefing, would improve OR team members’ perceived informedness and ESU. The intervention showed meaningful improvements in both domains for several professional groups. Informedness was significantly higher during the intervention for anaesthesia providers and circulators, ESU improved for anaesthesia providers, scrub technicians and circulators. No significant differences were observed for residents.

First, the study addressed the concern among OR team members about receiving information from surgeons during operations.^29^ By initiating the StOP?-protocol, where the surgeon addresses the entire team, the protocol appears instrumental in fostering informedness, particularly benefiting anaesthesia providers and circulators who may miss information due to their distance from the operative field. This aligns with the recognition that surgeons mainly communicate with those directly at the table.^30 52^ While self-reported informedness does not cover the full scope of SA, it reflects perceived access to critical information—a precondition for shared mental models, coordination and safe surgical action. It can therefore be regarded as a relevant indicator of teamwork-related processes in applied OR research.^19 53^ Our findings also align with the importance of a ‘talking to the room’ approach for maintaining shared understanding in medical and crisis teams.^54^

Second, the intervention may make speaking up easier, as evidenced by the increased perceived ESU among scrub technicians, anaesthesia providers and circulators. This is attributed to the explicit encouragement from the surgeon to voice questions or concerns as part of the StOP? protocol and aligns with studies demonstrating that even brief encouragements from team leaders increased voice behaviours.^39^ As the protocol mandates a pause in activities, it also creates a shared, structured moment for team contributions, reducing ambiguity about when it is an appropriate moment to provide input.

The benefits of the StOP?-protocol for informedness and ESU were not uniform across professions. Residents and scrubs, working directly at the table, did not feel more informed, and residents did not experience greater ESU, suggesting that OR teams are not single units, but may have to be seen as composed of different sub-teams.^55 56^ The intervention may also have levelled the communication field by benefiting lower-status roles.^57^

### Effects of operative access and difficulty of operation on feeling informed and ESU

Additional analyses explored potential influences of operative access and difficulty on feeling informed and ESU. Feeling informed was not higher for minimally invasive as compared with open procedures, despite the availability of monitors in the OR for minimally invasive operations. Anaesthesia providers actually reported feeling less informed during minimally invasive procedures. This could be due to the limitations of monitor information or inconsistent access to monitors. Probing whether the intervention had different effects for minimally invasive versus open operations for residents, scrubs or anaesthesia providers showed no significant effects. However, circulators reported higher informedness during open operations during the intervention phase. In all hospitals, trained scrub technicians worked either at the table or as circulators. During minimally invasive procedures, circulators thus had access to the ‘habitual’ monitor information, while during open procedures, they mostly had to rely on information from the surgeons; this may explain this effect.

Difficulty of operation was related to lower informedness of residents, scrub technicians and circulators, as well as to lower ESU for anaesthesia providers and circulators. This is important because SA certainly is crucial in non-standard situations,^20^ as is speaking up.^58^

Explanations for lower feelings of informedness during difficult operations may be twofold: difficult operations may be inherently harder for team members to follow, and they are also more challenging for surgeons to explain. Although the StOP?-protocol could, in principle, help close such informational gaps, it did not show a stronger effect for difficult operations. Prior research suggests that in stressful situations, individuals narrow their focus on their own tasks and lose the broader team perspective^59 60^; thus, surgeons may not have adjusted their communication to the team’s increased information needs during difficult operations.

Lower perceived ESU during more difficult operations indicates that speaking up is also a question of timing. Prior research showed that high workload and distressed colleagues reduce speaking up.^61 62^ Again, probing the interaction effects between difficulty and the intervention on ESU indicates that the StOP? protocol was not more effective in difficult operations.

### Limitations and strengths

Typical limitations of intervention studies apply. Participants could not be blinded, as the StOP? protocol inherently changes surgeon behaviour. As a before-after study, the design cannot eliminate all time-related confounding. However, the staggered roll-out across hospitals, the absence of major organisational or technical changes during the study period, comparable case-mix, operative access and perceived difficulty across periods (online supplemental table S1) and adjustments for hospital, operative access and difficulty all support the robustness of the findings. All outcomes were self-reported; however, questionnaires were completed at or near the end of each operation, response rates were very high, and the study included three hospitals, reducing the risk of systematic bias. Another limitation is that clustering could only be modelled for the main surgeon. Because other team members could not be consistently identified across operations, residual dependencies at the team level cannot be excluded. Although the study was conducted in Swiss hospitals only, the intervention targets general team-processes central to safe teamwork. Nevertheless, transferability to other cultural and healthcare settings cannot be assumed automatically, because local norms, cultures, hierarchies and workflow may influence effects.

The StOP? briefing was deliberately designed to fit routine operative workflow by being brief, procedure-adapted and timed to natural transition points during the operation, and to complement rather than replace existing safety routines such as the WHO surgical safety checklist.^63^

In conclusion, a short intraoperative briefing improved perceived informedness and ESU—two team-process outcomes relevant to safe surgical teamwork.^22 29 64^ As an alternative to the structured StOP?-protocol, simply encouraging surgeons to share more information during an operation may seem intuitive. However, challenges like limited attentional capacity,^65 66^ having to provide updates as a secondary task to the main task of operating,^67^ interruption of flow experience,^68^ and underestimation of information needs^29^ may prevent surgeons from spontaneously updating the team. Thus, simply encouraging more information sharing is unlikely to be sufficient.

The findings of this study suggest that implementing the StOP?-protocol alongside WHO checklists may be a useful way to support team informedness and perceived ESU in OR teams. While the present study examined the effects of the intervention, sustained implementation in routine practice depends on additional conditions. In routine practice, adherence to the protocol may be challenging because updating the team is an additional communicative task that must be actively initiated rather than being inherently prompted by the surgical workflow. Although adherence to the StOP?-protocol was very high in the present study, this figure was obtained under study conditions, with a research team member present in the OR, and therefore likely overestimates routine adherence. Thus, even for a brief and apparently simple intervention, conditions known from other implementation efforts are likely to matter, including local leadership support, integration into broader teamwork-related training,^69–71^ monitoring of adherence, periodic feedback and incorporation into routine OR workflows.

Taken together, the findings of this study suggest that the StOP?-protocol may be a promising addition to perioperative safety practices, with the potential to strengthen team informedness and ESU when thoughtfully integrated into local OR workflows.

## Supplementary material

10.1136/bmjoq-2025-003989 online supplemental file 1

## Data Availability

Data are available upon reasonable request.
