# Supplementary material for: Feeling better informed and more confident to speak up in the operating room: team effects of a structured intraoperative briefing intervention – a before-after study
Source: BMJ Open Qual. 2026 Oct 20;15(4):e003989. doi: 10.1136/bmjoq-2025-003989 (PMC13684205; doi:10.1136/bmjoq-2025-003989)
Supplement: Supplementary file 1 [file bmjoq-15-4-s001.docx]

**Supplementary Materials**

**Feeling better informed and more confident to speak up in the operating room: Team effects of a structured intraoperative briefing intervention. A before-after study.**

**Item provenance:**

***Feeling informed*** was assessed using two questions, combined into an index: “To what extent were you always informed about the plans and strategies the surgeons had in mind?” and “To what extent were you always informed about what the surgeons were doing at the very moment?”. These questions were adapted from the SART (Situation Awareness Rating Technique; Taylor, 1990; cf. Salmon et al., 2009), specifically from the item measuring information quantity, “How much information have you gained about the situation? Have you received and understood a great deal of knowledge (high) or very little? (low)”.

***Ease of speaking up*** (ESU) was assessed with the question “During this operation, could you have asked questions or raised issues at any time?”. This item was adapted from the *Speaking Up Climate for Professionalism* (SUC-Prof) scale, “In my clinical area, it is difficult to speak up if I observe unprofessional behaviour” (Martinez et al., 2015).

**Figure S1** *Instructions StOP? protocol for the surgical team*

**What is the StOP? protocol?**

- The StOP? protocol is a brief, structured, intraoperative briefing. The aim of the StOP? protocol is to inform everyone working in the operating room about the status of the operation and its further course.

**How is the StOP? protocol carried out?**

1. The surgeon interrupts the operation and announces the start of the StOP protocol loudly.

Everyone interrupts their work; anesthesiology providers and circulators approach the table.

2. The StOP? protocol follows this scheme:
   **St**atus:
   *The surgeon explains the current situation in a few sentences*

**O**bjectives:
*The surgeon states goals and next steps*
**P**roblems:
*The surgeon mentions any potential problems
that may be expected.*

**?** Questions:
*The surgeon asks if anyone has any questions or remarks*

Carrying out the StOP? protocol typically takes one to two minutes.

**Table S1** *Type of surgery, surgical access and surgeon difficulty ratings
across the baseline and intervention period.*

|  | **Baseline** | | **Intervention** | | **Total** | |
| --- | --- | --- | --- | --- | --- | --- |
|  | N | % | N | % | N | % |
| Total | 165 | 100.0% | 206 | 100.0% | 371 | 100.0% |
| **Type of surgery** ^b^ |  |  |  |  |  |  |
| Upper GI tract | 13 | 7.9% | 17 | 8.3% | 30 | 8.1% |
| Lower GI tract | 25 | 15.2% | 39 | 18.9% | 64 | 17.3% |
| Liver | 24 | 14.5% | 26 | 12.6% | 50 | 13.5% |
| Pancreas | 26 | 15.8% | 27 | 13.1% | 53 | 14.3% |
| Hernia | 17 | 10.3% | 22 | 10.7% | 39 | 10.5% |
| Cholecystectomy | 15 | 9.1% | 24 | 11.7% | 39 | 10.5% |
| Bypass or gastric sleeve | 16 | 9.7% | 15 | 7.3% | 31 | 8.4% |
| Kidney transplant | 8 | 4.8% | 9 | 4.4% | 17 | 4.6% |
| Thoracic surgery | 5 | 3.0% | 6 | 2.9% | 11 | 3.0% |
| Vascular surgery | 4 | 2.4% | 6 | 2.9% | 10 | 2.7% |
| Other procedure | 12 | 7.3% | 15 | 7.3% | 27 | 7.3% |
| **Surgical access** ^c^ |  |  |  |  |  |  |
| open or converted | 93 | 56.4% | 110 | 53.4% | 203 | 54.7% |
| laparoscopic | 72 | 43.6% | 96 | 46.6% | 168 | 45.3% |
| **Difficulty (main surgeon)^a^** |  |  |  |  |  |  |
| Low difficulty (1-4) | 82 | 49.7% | 108 | 53.5% | 190 | 51.8% |
| High difficulty (5-7) ^d^ | 83 | 50.3% | 94 | 46.5% | 177 | 48.2% |
| Difficulty, scale ^b^ | 165 | 4.33 (SD=1.6) | 202 | 4.32 (SD=1.6) | 367 | 4.32 (SD=1.6) |
| ^a^ Available for 367 surgeries  Differences between baseline and intervention:  ^b^ Type of surgery: Chi2(df=10)= 2.83, P=0.99;  ^c^ Surgical access: Chi2(df=1)=0.325, P=.57;  ^d^ Difficulty, dichotomized: Chi2(df=1)=0.517, P=0.47  ^e^ Difficulty, scale: T=0.039, df = 365, P=0.97  SD = standard deviation | | | | | | |

**Table S2** *Correlation heatmap between feeling informed and ease of speaking up within and between residents, scrubs, anesthesia providers and circulators.*

**Table S3** *Feeling informed, separately for each profession: Results of the ANCOVA, including interaction effects between access and intervention and difficulty and intervention*

|  | **FEELING INFORMED (ANCOVA MODELS)** | | | | | | | | | | | | |
| --- | --- | --- | --- | --- | --- | --- | --- | --- | --- | --- | --- | --- | --- |
|  |  | between subject effects | | | | | | parameter estimates | | | | | |
| **Profession** | Source | | df | | MS | F | B | | SE | t | P | CI lo | CI hi |
| **Residents** | Intercept | | 1 | 475.529 | | 527.804 | 6.593 | | 0.293 | 22.530 | >0.001 | 6.017 | 7.169 |
|  | Intervention (reference: intervention) | | 1 | 0.083 | | 0.092 | -0.034 | | 0.112 | -0.304 | 0.761 | -0.225 | 0.187 |
|  | Access (0 = minimal) | | 1 | 0.477 | | 0.530 | 0.088 | | 0.121 | 0.728 | 0.467 | -0.150 | 0.327 |
|  | Difficulty | | 1 | 6.215 | | 6.898 | -0.092 | | 0.035 | -2.626 | 0.009 | -0.160 | -0.023 |
|  | Hospital 1 | | 1 | 1.102 | | 1.223 | 0.161 | | 0.145 | 1.106 | 0.270 | -0.125 | 0.447 |
|  | Hospital 2 | | 1 | 0.588 | | 0.653 | -0.117 | | 0.145 | -0.808 | 0.420 | -0.401 | 0.168 |
|  | access * intervention | | 1 | 0.924 | | 1.026 | -0.227 | | 0.224 | -1.013 | 0.312 | -0.667 | 0.214 |
|  | difficulty * intervention | | 1 | 1.322 | | 1.470 | 0.079 | | 0.065 | 1.212 | 0.226 | -0.049 | 0.208 |
| **Scrubs** | Intercept | | 1 | 528.490 | | 504.550 | 6.398 | | 0.286 | 22.358 | >0.001 | 5.836 | 6.961 |
|  | Intervention (reference: intervention) | | 1 | 1.752 | | 1.673 | -0.140 | | 0.109 | -1.293 | 0.197 | -0.354 | 0.073 |
|  | Access (0 = minimal) | | 1 | 1.794 | | 1.713 | 0.153 | | 0.117 | 1.309 | 0.191 | -0.077 | 0.383 |
|  | Difficulty | | 1 | 9.192 | | 8.776 | -0.104 | | 0.035 | -2.962 | 0.003 | -0.173 | -0.035 |
|  | Hospital 1 | | 1 | 0.101 | | 0.096 | -0.042 | | 0.136 | -0.310 | 0.757 | -0.309 | 0.225 |
|  | Hospital 2 | | 1 | 1.490 | | 1.423 | -0.170 | | 0.143 | -1.193 | 0.234 | -0.451 | 0.110 |
|  | access * intervention | | 1 | 0.173 | | 0.165 | 0.088 | | 0.217 | 0.106 | 0.685 | -0.339 | 0.515 |
|  | difficulty * intervention | | 1 | 0.011 | | 0.011 | 0.007 | | 0.066 | 0.104 | 0.917 | -0.123 | 0.137 |
| **Anesthesia** | Intercept | | 1 | 484.687 | | 328.373 | 6.452 | | 0.341 | 18.907 | >0.001 | 5.781 | 7.123 |
|  | Intervention (reference: intervention) | | 1 | 47.079 | | 31.896 | -0.727 | | 0.129 | -5.648 | >0.001 | -0.981 | -0.474 |
|  | Access (0 = minimal) | | 1 | 9.795 | | 6.636 | -0.359 | | 0.139 | -2.576 | 0.010 | -0.633 | -0.085 |
|  | Difficulty | | 1 | 5.510 | | 3.733 | -0.081 | | 0.042 | -1.932 | 0.054 | -0.163 | 0.001 |
|  | Hospital 1 | | 1 | 0.003 | | 0.002 | -0.007 | | 0.161 | -0.046 | 0.963 | -0.325 | 0.310 |
|  | Hospital 2 | | 1 | 0.316 | | 0.214 | -0.078 | | 0.169 | -0.463 | 0.644 | -0.410 | 0.254 |
|  | access * intervention | | 1 | 1.182 | | 1.1004 | -0.258 | | 0.258 | -1.002 | 0.317 | -0.765 | 0.248 |
|  | difficulty * intervention | | 1 | 1.046 | | 0.708 | 0.066 | | 0.079 | 0.842 | 0.401 | -0.088 | 0.221 |
| **Circulators** | Intercept | | 1 | 510.931 | | 372.323 | 6.510 | | 0.328 | 19.849 | >0.001 | 5.865 | 7.156 |
|  | Intervention (reference: intervention) | | 1 | 28.286 | | 20.613 | -0.564 | | 0.124 | -4.540 | >0.001 | -0.808 | -0.320 |
|  | Access (0 = minimal) | | 1 | 1.344 | | 0.979 | 0.133 | | 0.134 | 0.990 | 0.323 | -0.131 | 0.396 |
|  | Difficulty | | 1 | 19.927 | | 14.521 | -0.153 | | 0.040 | -3.811 | >0.001 | -0.232 | -0.074 |
|  | Hospital 1 | | 1 | 0.931 | | 0.678 | -0.128 | | 0.156 | -0.823 | 0.411 | -0.434 | 0.178 |
|  | Hospital 2 | | 1 | 18.670 | | 13.605 | -0.601 | | 0.163 | -3.688 | >0.001 | -0.922 | -0.281 |
|  | access * intervention | | 1 | 12.529 | | 9.343 | 0.75 | | 0.245 | 3.054 | 0.002 | 0.268 | 1.233 |
|  | difficulty * intervention | | 1 | 0.201 | | 0.146 | -0.029 | | 0.076 | -0.382 | 0.703 | -0.178 | 0.12 |

**Notes**: B values represent adjusted regression coefficients from ANCOVA models. Coefficients for the intervention variable reflect SPSS reference coding, with the intervention condition as the reference category; negative values therefore indicate higher scores in the intervention compared to baseline. Feeling informed was measured on a 7-point scale, with higher values indicating greater feeling of being informed. Access was coded as 0 = minimally invasive and 1 = open procedure. Difficulty was treated as a continuous variable, with higher values indicating greater perceived difficulty. Hospital variables represent dummy-coded comparisons with the reference hospital. df = degrees of freedom, MS = mean square, B=unstandardized coefficient, SE = standard error, CI = 95% confidence interval, lo = low, hi = high

Main effects are reported based on the ANCOVA model without interaction terms.

**Figure** *S2 Feeling informed during low versus high difficult operations, based on adjusted values*

| **a** Feeling informed residents low vs hi difficult operations | **b** Feeling informed scrubs low vs high difficult operations |
| --- | --- |
|   P=0.009 |   P=0.003 |
| **c** Feeling informed Anesthesia low vs high difficult operations | **d** Feeling informed Circulators low vs high difficult operations |
|   P=0.054 |   P<0.001 |

**Notes:** Based on predicted values, adjusted for hospital and operative access. Feeling informed is based on self-reports, provided immediately after the operation (1-7).

**Figure** *S3 Illustration of the interaction effect of feeling informed for open and minimally invasive procedures during baseline and intervention for circulators*

Circulators: Feeling informed in open and minimally invasive operations during baseline and intervention

|  | Baseline | Intervention |
| --- | --- | --- |
| open | 4.996 | 5.896 |
| minimally invasive | 5.554 | 5.695 |

**Table S4** *Ease of speaking up, separately for each profession: Results of the ANCOVA, including interaction effects between access and intervention and difficulty and intervention*

|  | **EASE OF SPEAKING UP (ANCOVA MODELS)** | | | | | | | | | | |
| --- | --- | --- | --- | --- | --- | --- | --- | --- | --- | --- | --- |
|  |  | between subject effects | | | | parameter estimates | | | | | |
| **Profession** | Source | df | MS | F | B | SE | t | P | CI lo | CI hi |  |
| **Residents** | Intercept | 1 | 485.911 | 214.806 | 6.712 | 0.469 | 14.308 | 0.000 | 5.788 | 7.635 |  |
|  | Intervention (reference: intervention) | 1 | 0.007 | 0.003 | 0.010 | 0.178 | 0.057 | 0.955 | -0.341 | 0.361 |  |
|  | Access (0 = minimal) | 1 | 2.407 | 1.064 | -0.200 | 0.194 | -1.031 | 0.303 | -0.582 | 0.182 |  |
|  | Difficulty | 1 | 0.006 | 0.002 | 0.003 | 0.056 | 0.049 | 0.961 | -0.107 | 0.112 |  |
|  | Hospital 1 | 1 | 2.053 | 0.908 | -0.220 | 0.231 | -0.953 | 0.342 | -0.675 | 0.235 |  |
|  | Hospital 2 | 1 | 27.255 | 12.048 | -0.799 | 0.230 | -3.471 | 0.001 | -1.251 | -0.346 |  |
|  | access * intervention | 1 | 0.039 | 0.017 | -0.047 | 0.357 | -0.132 | 0.895 | -0.749 | 0.655 |  |
|  | difficulty * intervention | 1 | 2.998 | 1.327 | 0.119 | 0.104 | 1.152 | 0.250 | -0.085 | 0.323 |  |
| **Scrubs** | Intercept | 1 | 515.292 | 279.380 | 6.447 | 0.381 | 16.934 | 0.000 | 5.698 | 7.196 |  |
|  | Intervention (reference: intervention) | 1 | 12.206 | 6.618 | -0.371 | 0.144 | -2.573 | 0.010 | -0.655 | -0.087 |  |
|  | Access (0 = minimal) | 1 | 2.781 | 1.508 | 0.191 | 0.156 | 1.228 | 0.220 | -0.115 | 0.497 |  |
|  | Difficulty | 1 | 5.507 | 2.986 | -0.080 | 0.047 | -1.728 | 0.085 | -0.172 | 0.011 |  |
|  | Hospital 1 | 1 | 0.118 | 0.064 | 0.045 | 0.180 | 0.253 | 0.801 | -0.308 | 0.399 |  |
|  | Hospital 2 | 1 | 7.456 | 4.043 | -0.381 | 0.190 | -2.011 | 0.045 | -0.754 | -0.008 |  |
|  | access * intervention | 1 | 2.298 | 1.247 | 0.321 | 0.288 | 1.117 | 0.265 | -0.245 | 0.887 |  |
|  | difficulty * intervention | 1 | 0.245 | 0.132 | 0.032 | 0.088 | 0.364 | 0.716 | -0.141 | 0.204 |  |
| **Anesthesia** | Intercept | 1 | 541.367 | 335.927 | 6.606 | 0.357 | 18.528 | 0.000 | 5.905 | 7.307 |  |
|  | Intervention (reference: intervention) | 1 | 10.546 | 6.544 | -0.344 | 0.135 | -2.558 | 0.011 | -0.609 | -0.080 |  |
|  | Access (0 = minimal) | 1 | 1.631 | 1.012 | -0.146 | 0.146 | -1.006 | 0.315 | -0.433 | 0.140 |  |
|  | Difficulty | 1 | 7.059 | 4.380 | -0.091 | 0.044 | -2.093 | 0.037 | -0.177 | -0.006 |  |
|  | Hospital 1 | 1 | 3.517 | 2.182 | 0.249 | 0.169 | 1.477 | 0.140 | -0.083 | 0.581 |  |
|  | Hospital 2 | 1 | 0.024 | 0.015 | -0.021 | 0.176 | -0.122 | 0.903 | -0.368 | 0.325 |  |
|  | access * intervention | 1 | 0.335 | 0.207 | 0.123 | 0.269 | 0.455 | 0.649 | -0.407 | 0.653 |  |
|  | difficulty * intervention | 1 | 0.623 | 0.386 | 0.051 | 0.082 | 0.621 | 0.535 | -0.111 | 0.213 |  |
| **Circulators** | Intercept | 1 | 545.617 | 364.877 | 6.621 | 0.344 | 19.236 | 0.000 | 5.944 | 7.298 |  |
|  | Intervention (reference: intervention) | 1 | 6.977 | 4.666 | -0.281 | 0.130 | -2.160 | 0.031 | -0.537 | -0.025 |  |
|  | Access (0 = minimal) | 1 | 3.660 | 2.448 | 0.220 | 0.141 | 1.565 | 0.119 | -0.057 | 0.496 |  |
|  | Difficulty | 1 | 10.437 | 6.980 | -0.111 | 0.042 | -2.642 | 0.009 | -0.194 | -0.028 |  |
|  | Hospital 1 | 1 | 7.841 | 5.243 | -0.374 | 0.164 | -2.290 | 0.023 | -0.696 | -0.053 |  |
|  | Hospital 2 | 1 | 24.101 | 16.117 | -0.687 | 0.171 | -4.015 | 0.000 | -1.023 | -0.350 |  |
|  | access * intervention | 1 | 4.479 | 3.012 | 0.45 | 0.259 | 1.736 | 0.084 | -0.06 | 0.959 |  |
|  | difficulty * intervention | 1 | 0.005 | 0.004 | -0.005 | 0.079 | -0.06 | 0.925 | -0.16 | 0.151 |  |

**Notes**: B values represent adjusted regression coefficients from ANCOVA models. Coefficients for the intervention variable reflect SPSS reference coding, with the intervention condition as the reference category; negative values therefore indicate higher scores in the intervention compared to baseline. Ease of speaking up was measured on a 7-point scale, with higher values indicating greater ease of speaking up. Access was coded as 0 = minimally invasive and 1 = open procedure. Difficulty was treated as a continuous variable, with higher values indicating greater perceived difficulty. Hospital variables represent dummy-coded comparisons with the reference hospital. df = degrees of freedom, MS = mean square B=unstandardized coefficient, SE = standard error, CI = 95% confidence interval, lo = low, hi = high

Main effects are reported based on the ANCOVA model without interaction terms.

**Figure S4 *Ease of speaking up during low- versus high-difficulty operations, based on adjusted values***

| **a** ESU residents: low- vs high-difficulty operations | **b** ESU scrubs: low- vs high-difficulty operations |
| --- | --- |
|   P=0.961 |   P=0.085 |
| **c** ESU anesthesia: low- vs high-difficulty operations | **d** ESU circulators: low- vs high-difficulty operations |
|   P=0.037 |   P=0.009 |

**Notes:** Based on predicted values, adjusted for hospital and operative access. Ease of speaking up is based on self-reports, provided immediately after the operation (1-7).

**Table S5. *Robustness check:* *Comparison of ANCOVA and mixed-effects models for intervention effects***

| Outcome | Profession | ANCOVA | | Mixed model including random intercepts for surgeons | | |
| --- | --- | --- | --- | --- | --- | --- |
| **Feeling informed** |  | B | P | B | P | Adjusted ICC |
|  | Residents | -0.034 | 0.761 | 0.014 | 0.909 | 0.05 |
|  | Scrub technicians | -0.140 | 0.197 | -0.104 | 0.369 | 0.06 |
|  | Anesthesia providers | -0.727 | <0.001 | -0.683 | <0.001 | 0.08 |
|  | Circulators | -0.564 | <0.001 | -0.520 | <0.001 | 0.04 |
| **Ease of speaking up** |  |  |  |  |  |  |
|  | Residents | 0.010 | 0.955 | 0.113 | 0.537 | 0.00 |
|  | Scrub technicians | -0.371 | 0.010 | -0.345 | 0.020 | 0.08 |
|  | Anesthesia providers | -0.344 | 0.011 | -0.302 | 0.033 | 0.14 |
|  | Circulators | -0.281 | 0.031 | -0.312 | 0.020 | 0.05 |

**Notes**: Coefficients for the intervention variable reflect SPSS reference coding, with the intervention condition as the reference category; negative values therefore indicate higher scores in the intervention compared to baseline. The adjusted ICC indicates the proportion of residual variance attributable to differences between surgeons after controlling for the covariates in the model.

Martinez, W., Etchegaray, J. M., Thomas, E. J., Hickson, G. B., Lehmann, L. S., Schleyer, A. M., . . . Bell, S. K. (2015). 'Speaking up' about patient safety concerns and unprofessional behaviour among residents: validation of two scales. *BMJ Qual Saf, 24*(11), 671-680. doi:10.1136/bmjqs-2015-004253

Salmon, P. M., Stanton, N. A., Walker, G. H., Jenkins, D., Ladva, D., Rafferty, L., & Young, M. (2009). Measuring Situation Awareness in complex systems: Comparison of measures study. *International Journal of Industrial Ergonomics, 39*(3), 490-500. doi:10.1016/j.ergon.2008.10.010
